# Effect of long term high altitude exposure on cardiovascular autonomic adjustment during rest and post-exercise recovery

**DOI:** 10.1186/s40557-018-0240-1

**Published:** 2018-05-11

**Authors:** Prem Bhattarai, Bishnu H. Paudel, Dilip Thakur, Balkrishna Bhattarai, Bijay Subedi, Rita Khadka

**Affiliations:** 1 Department of Physiology, Birat Medical College and Teaching Hospital, Tankisiniwari Morang, Nepal; 2 Department of Basic and Clinical Physiology, B.P Koirala Institute of Health Sciences, Dharan, Nepal; 3 Department of Anaesthesiology and Critical Care, B.P Koirala Institute of Health Sciences, Dharan, Nepal; 4 Department of Anatomy and Physiology, Pokhara University, Kaski, Nepal

**Keywords:** Highlanders, Heart rate variability, Exercise

## Abstract

**Background:**

Despite the successful adaptation to high altitude, some differences do occur due to long term exposure to the hypoxic environment. The effect of long term high altitude exposure on cardiac autonomic adjustment during basal and post-exercise recovery is less known. Thus we aimed to study the differences in basal cardiac autonomic adjustment and its response to exercise in highlanders and to compare it with lowlanders.

**Methods:**

The study was conducted on 29 healthy highlander males who were born and brought up at altitude of 3000 m and above from the sea level, their cardiac autonomic adjustment was compared with age, sex, physical activity and ethnicity-matched 29 healthy lowlanders using Heart Rate Variability (HRV) during rest and recovery from sub-maximal exercise (3 m step test). Intergroup comparison between the highlanders and lowlanders and intragroup comparison between the rest and the postexercise recovery conditions were done.

**Results:**

Resting heart rate and HRV during rest was comparable between the groups. However, heart rate recovery after 3 min step test was faster in highlanders (*p* < 0.05) along with significantly higher LF power and total power during the recovery phase. Intragroup comparison of highlanders showed higher SDNN (*p* < 0.05) and lower LF/HF ratio (*p* < 0.05) during recovery phase compared to rest which was not significantly different in two phases in lowlanders. Further highlander showed complete recovery of RMSSD, NN50, pNN50 and HF power back to resting level within five minutes, whereas, these parameters failed to return back to resting level in lowlanders within the same time frame.

**Conclusion:**

Highlanders completely recovered back to their resting state within five minutes from cessation of step test with parasympathetic reactivation; however, recovery in lowlanders was delayed.

**Electronic supplementary material:**

The online version of this article (10.1186/s40557-018-0240-1) contains supplementary material, which is available to authorized users.

## Introduction

Many people live and work at high altitude hypoxic environment without any apparent adverse effects. Despite the successful adaptation to high altitude in permanent residents, time dependent changes occur to autonomic nervous system [1]. Sympathetic stimulation occurs with acute or sub- acute altitude exposure followed by progressive blunting of the sympathetic response with prolonged hypoxic exposure [1–3]. There are chronic hypoxia-induced autonomic changes, coupled with a possible reduction in intrinsic heart rate, decreased resting heart rate and increased heart rate variability at rest [3]. However, effects of years to generations of high-altitude exposure on the cardiac autonomic modulation during rest, exercise and post exercise recovery is still conflicting and less attention is drawn in comparing the cardiovascular autonomic adjustment of these highlanders with their low lander counterparts of similar age group and physical activity.

Heart rate variability (HRV) is variations in instantaneous heart rate or R-R intervals, recognized as a powerful tool for the estimation of cardiac autonomic modulation [4]. During physical exercise heart rate is increased which is associated with parasympathetic withdrawal and sympathetic activation. The rate of decrease in heart rate and increased variability after physical exercise can be used as a powerful index of cardiac vagal reactivation.

Accordingly, the study is designed to find the difference in autonomic adjustment in highlanders from that of lowlanders during rest and post-exercise recovery.

## Methods

### Subjects

After obtaining ethical clearance from Institute’s Ethical Review Board (IERB) of B.P Koirala Institute of Health Sciences the study was conducted on 29 healthy male subjects born and brought up at the altitude of 3000 m and above from the sea level. Their cardiac autonomic adjustment was compared with age, sex, physical activity and ethnicity-matched healthy lowlanders who were born and brought up at the altitude less than 500 m above the sea level. Physical activity matching of the subjects was done using the history of weekly physical activity performed by the subjects based on the physical activity rating (PAR) questionnaire presented in Additional file 1. The subjects were selected using non-probability purposive sampling technique using below mentioned inclusion and exclusion criteria.

### Inclusion criteria

#### Highlander subjects

- Consenting healthy males of age ranging from 15 to 46 years who were born and brought up at high altitude (3000 m above the sea level).

#### Lowlander subjects

- Consenting healthy males who were born and brought up in the lowland, altitude of less than 500 m above the sea level and age, ethinicity and physical activity matched with highlanders.

### Exclusion criteria for subjects

1. Subjects with any neuromuscular and autonomic disorders.
2. Persons with clinically diagnosed hypertension, diabetes, cardiovascular diseases etc.
3. Subjects on any medication.
4. Subjects with drug dependence.
5. Subjects with obesity(BMI > 25 kg/m^2^) and malnutrition (BMI < 18 kg/m^2^)
6. For smokers and alcohol drinkers, the subjects with scores of Fagerstrom test for nicotine ndependence ≥4 and alcohol use disorder identification (AUDIT) ≥ 8 were excluded.

### Study location

All the studies on highlander subjects were performed at the lab that was set at Gufapokhari, Sankhuwashaba, Nepal (2960 m from sea level). The similar study was performed for lowlanders in neurophysiology lab of Department of Basic and Clinical Physiology at BPKIHS, Dharan, Nepal (350 m from sea level).

### Recording of anthropometric variables

Anthropometric and cardio respiratory variables including resting heart rate were recorded using standard protocol.

### Recording of resting HRV

For resting HRV, resting cardiac cycle (R-R interval) signals at spontaneous respiration were recorded for 5 min in the supine position after 15 min of supine rest. The R-R interval for HRV was sampled using portable POLAR HEART RATE MONITOR (S810i) USA/GBR. The elastic strap with electrode was moistened and applied to the chest. The transmitter attached to electrode was firmly and comfortably held in central upright position. The wrist receiver was placed within 3 ft or 1 m from the transmitter and assured that the subject was not near to high voltage power lines, television, mobile phones or other sources of electromagnetic disturbance. Room temperature was maintained at 21 ± 2 °C.

### Three minutes step test and recovery heart rate

Subjects were asked to perform submaximal, 3 min step test on the bench of 30 cm height for three minutes at the rate of 96 steps per minute [5]. Pulse rate of the first minute immediately after stopping of step test was recorded as recovery heart rate.

### Calculation physical fitness score

Physical fitness score of all the subjects was calculated from recovery heart rate based on Young Men’s Christian Association (YMCA) guidelines [6].

Here are the age-adjusted standards based on guidelines published by YMCA.

**Table:** 

	18–25	26–35	36–45	46–55	56–65	65+
Excellent	50–76	51–76	49–76	56–82	60–77	59–81
Good	79–84	79–85	80–88	87–93	86–94	87–92
Above Average	88–93	88–94	92–88	95–101	97–100	94–102
Average	95–100	96–102	100–105	103–111	103–109	104–110
Below Average	102–107	104–110	108–113	113–119	111–117	114–118
Poor	111–119	114–121	116–124	121–126	119–128	121–126
Very Poor	124–157	126–161	130–163	131–159	131–154	130–151

Fitness score was assigned to subjects based on 1-min post exercise recovery heart rate count for Men, Based on Age defined by YMCA guidelines.

- Fitness score 1: Very poor
- Fitness score 2: Poor category
- Fitness score 3: Below average
- Fitness score 4: Average
- Fitness score 5: Above average
- Fitness score 6: Good
- Fitness score 7: Excellent

### Recording of recovery HRV

After the completion of 3 min step test, subjects were asked to rest in the supine position and recording of recovery RR intervals were taken for 5 min using portable POLAR HEART RATE MONITOR (S810i) USA/GBR.

### Analysis of HRV

The R-R intervals recorded in the Polar device were transmitted to the computer using Polar Precision Performance software. Processed RR interval were analyzed for time domain and frequency domain measures using Kubios HRV (version 2.1, Kuopio FINLAND).

Time domain measures include SDNN (standard deviation of normal to normal RR intervals), RMSSD (root mean square of the difference of successive RR intervals), NN50 count (number of RR intervals that differ more than 50 ms), pNN50 (percentage of consecutive RR intervals that differs from more than 50 ms).

Frequency domain analysis was done using Fast Fourier transformation. Frequency domain measures included power of Low frequency (LF, frequency between 0.04 and 0.15 Hz), High frequency (HF, frequency between 0.15 and 0.4 Hz), LF n.u and HF n.u (the power of low frequency and high frequency in normalized unit) and LF/HF (ratio of LF and HF power).

### Statistical analysis

HRV results were exported to Microsoft Excel and then to SPSS, version 11.5 for further analysis. Normally distributed data were expressed as Mean ± S.D. and non-normally distributed data as Median (Interquartile range). Comparisons between the groups and intra group comparisons were made by paired t-test for normally distributed data and Wilcoxon signed rank for non-normally distributed data.

## Results

### Comparison of anthropometric and cardiorespiratory variables

There were no significant differences in age, weight and body mass index (BMI) between highlander and lowlander groups, however, mean height of highlanders was shorter than lowlanders. Systolic blood pressure (SBP) and Diastolic blood pressure (DBP) were significantly higher in highlanders. Resting heart rate was comparable between the groups, however, recovery heart rate after cessation of step test was significantly lower in highlanders compared to lowlanders. The data are presented in Table 1.

**Table 1:** Comparison of anthropometric and cardiorespiratory variables

S.N	Variable	Highlanders (*n* = 29) Mean ± SD	Lowlanders(*n* = 29) Mean ± SD	*p*-value
1	Age (years)	28.76 ± 8.43	28.86 ± 8.21	NS
2	Age range (years)	17–46	16–45	
3	Height (cms)	1.65 ± 0.05	1.71 ± 0.06	0.001
4	Weight(Kgs)	63.38 ± 5.64	66.76 ± 6.78	NS
5	BMI (Kg/m^2^)	23.19 ± 1.89	22.84 ± 1.90	NS
6	Systolic BP (mmHg)	120.76 ± 10.40	115.52 ± 9.95	0.016
7	Diastolic BP (mmHg)	82.90 ± 8.12	78.14 ± 7.14	0.02
8	Resting HR (bpm)	67.83 ± 9.89	69.24 ± 7.99	NS
9	Recovery HR (bpm)	96.97 ± 18.44	106.28 ± 15.57	0.048
10	Fitness Score	4.45 ± 1.88	3.27 ± 1.75	0.00
11	Physical activity rating^a^	6.24 ± 0.87	5.98 ± 1.08	NS

The *p* < 0.05 was considered statistically significant

*BMI* Body Mass Index, *NS* statistically non-significance, *BP* Blood Pressure, *HR* Heart rate, *bpm* beats per minute

^a^ physical activity rating was calculated using questionare based on Jackson, et. al [16]

### Comparison of HRV between highlanders and lowlanders

All measures of Heart rate variability during rest were comparable between the groups. However, during recovery frequency domain measures, LF power and the total power were found significantly higher in highlanders compared to lowlanders whereas, other parameters of time domain and frequency domain were comparable between the groups. The results are presented in Table 2.

**Table 2:** Comparision of the parameters for HRV in the subjects

	Resting		Recovering	
Variable	Highlanders	Lowlanders	Highlanders	Lowlanders
Time domain				
SDNN^b^	**56.6(43–71.9)**	50.8(35.1–80.7)	**80.3(54.2–99.8)**	62.7 (50.7–79)
RMSSD^c^	47.3 (36.5–69)	**37.9 (31.6–81.6)**	48 (32.6–85.6)	**32.2 (23.5–53.3)**
NN50^c^	63 (36–142)	**63 (32–115)**	81 (35–138)	**27(11–75)**
pNN50%^c^	17.6(8.9-41.3)	**18 (9–39)**	22 (9.6–40.4)	**6.3 (2.3–17.4)**
Frequency domain				
LF power^a^	720 (450–1162)	763 (328–1262)	**758 (446–2005)**	**383 (182–740)**
HF power^c^	684(273–1268)	**498 (278–1035)**	487 (206–2048)	**208 (63–798)**
LF	56.1 (43.3–62.9)	54.5 (37.9–69.5)	56.7 (46.7–74.5)	63.9 (46.8–75.9)
HF	43.9 (37.1–56.7)	45.5 (30.5–62.1)	43.3 (25.5–53.3)	36.1 (24.1–53.2)
Total power^a^	2659(1397–4528)	2199(1453-6124)	**3662 (2232–6380)**	**1862 (860–3699)**
LF/HF^b^	**1.28 (0.76–1.69)**	1.30 (0.71–2.28)	**1.18 (.87–2.92)**	1.77 (0.88–3.14)

^a^significant different in HRV during recovery between highlanders and lowlanders

^b^significant different in highlanders between resting and recovery

^c^significant different in lowlanders between resting and recovery

Bold numbers indicate that the values are statistically significant

### Intragroup comparison of HRV in highlanders between rest and recovery

All the time domain measures recovered back to their resting level within five minutes after the completion of the step test with further increment in the SDNN value during recovery. All frequency domain measures recovered to resting level except LF/HF ratio which was significantly lower during recovery compared to resting state. The results are presented in Table 2.

### Intragroup comparison of HRV in lowlanders between rest and recovery

Time domain measures RMSSD, NN50 and pNN50 did not recover to their resting level within five minutes after the completion of step test however, SDNN recovered to resting condition. Frequency domain parameters except HF power recovered back to resting level after exercise with in five minutes however, HF power did not recover to resting level. The results are presented in Table 2.

## Discussion

This study was conducted to find out the differences in cardiovascular autonomic modulation of highlanders and lowlander during the rest and recovery after submaximal exercise. Our observation showed comparable basal heart rate but significantly speedy recovery of heart rate after step test in highlanders. The fitness score calculated based on the recovery heart rate was higher in highlanders compared to lowlanders although the physical activity rating of both study group was almost matched. The results of our study is similar to the study done by Boushel et al [7] where they studied the effect of high altitude acclimatization in resting and recovery heart rate. The speedy recovery in highlanders in the present study may be due to enhanced parasympathetic neural tone. Some earlier studies by Malohtara et al [8] and Sharshenova et al [9] showed the overall parasympathetic dominance in highlanders compared to lowlanders. The study done by Sharshenova et al [9] 2006 found higher SDNN, HF power and total power, indicating increased overall variability or parasympathetic modulation with increasing altitude in resting condition. In contrast to earlier studies, this study showed some peculiar result where the resting heart rate (HR) in both groups were comparable indicating similar type of sympathovagal balance, which is further supported by comparable results of HRV during rest.

Futher HRV spectral analysis during recovery showed increased LF power and total power in highlanders compared to lowlanders while rest of the variables of time domain (SDNN, RMSSD, NN50 and pNN50) and frequency domain measures (HF power, LF nu, HF nu, LF/HF ratio) were comparable among the groups. The result of total power indicated the increased overall variability of HRV during the recovery in highlander group. Some controversy is in the interpretation of LF component, which is considered by some to be the marker of sympathetic modulation and by others as the parameter that includes sympathetic, vagal and baroreflex influences [4, 10, 11]. LF component of HRV during recovery is predominantly influenced by changes of parasympathetic activity directly (through alterations of vagal cardiac activity causing fluctuations in LF band) and or indirectly (through changes of baroreflex sensitivity) [12]. Thus both the increased total power and LF power in our result can be interpreted in the same line as increased parasympathetic reactivation during recovery of highlanders compared to lowlanders. The result can also be correlated with the speedy recovery of heart rate in highlanders compared to lowlanders supporting our claim of increased parasympathetic reactivation.

The sympatho-vagal balance in highlanders was regained back within five minutes of recovery period to their resting state as shown by HRV. Further SDNN was increased while the LF/HF ratio was reduced indicating profound parasympathetic reactivation during the recovery phase. The result of our study were in agreement with the study done by Kluess et al [13] and Oida et al [14] suggesting that heart rate decay is carried out not by a withdrawal of the sympathetic pathway but by sympatho-vagal cooperation: both divisions are increasing their activities in reciprocal operations, shifting their balance slightly towards the vagal influence in the course of heart recovery.

HRV measure in lowlanders during recovery was considerably different from that of highlanders. Time domain measures, RMSSD, NN50 and pNN50 and HF power of frequency domain did not recover to the resting level within five minutes of recovery. The result of lowlanders is in contrast to highlander group indicating the poor recovery after step test, especially the reduction in parasympathetic activity persisted for longer in lowlanders. The results are in agreement with the finding of delayed heart rate recovery in lowlanders compared to highlanders.

These findings showing the similar type of sympatho-vagal adjustment during rest but increased parasympathetic dominance during recovery in highlanders could be interpreted as increased cardiovascular tolerance to exercise in them resulting from lifelong exposure to high altitude hypoxic environment. The result of our study suggests the unique nature of cardiac autonomic modulation where post-exercise recovery is found to be more influenced by high altitude exposure compared to basal adjustment.

There were several limitations of the study including the small sample size due to less number of inhabitants in the high altitude which can be rectified in future studies by conducting the study in different high altitude populations. Similarly different other parameters of our interest including absolute measurement of Vo2 max, measurement of maximum exercising capacity, estimation of hemoglobin concentration and blood gas analysis of all the highlanders and low lander subject could not be done.

Beside the number of limiatation and resources constrains our study have explored some important facts which have great importance in public health and clinical medicine, further this can provide the baseline data for future studies in this direction. One of the important finding was higher systolic and diastolic blood pressure in highlanders of this region compared to lowlanders which was similar to the earlier finding by Otsuka et al [15] where they found steeper increase in both systolic and diastolic blood pressure with age in highlanders compared to low landers. However, the result of our study could not be generalized because of lower sample size in our study but extensive study with larger sample size is recommended to explore the differences in normal range of blood pressure in different altitude conditions. The studies in this area will have a great application in clinical medicine practice in diagnosis and management of hypertension in high altitude population. Further the unique results of our study showing similar basal heart rate but better parasympathetic reactivation indicating better physical fitness in highlanders may be applied for better endurance training of different sports and military activities. The study on the other hand proves the heart rate recovery after exercise to be a useful tool to assess the cardiovascular fitness as the findiing of heart rate recovary and heart rate variability during recovery are indicating similar way of cardiovascular autonomic adjustment.

## Conclusions

The cardiac autonomic modulation shown by Heart Rate Variability during rest was similar in highlanders and lowlanders. However, highlanders completely recovered back to their resting condition within five minutes after the exercise, whereas, lowlanders failed to do so. This shows the unique nature of cardiac autonomic adjustment in highlanders where post-exercise recovery is found to be influenced more by high altitude exposure compared to basal adjustment.

## Additional file

Additional file 1: Physical Activity Rating (PAR) Questionnaire. (DOCX 12 kb)
