# Supplementary material for: Effect of long term high altitude exposure on cardiovascular autonomic adjustment during rest and post-exercise recovery
Source: Ann Occup Environ Med. 2018 May 11;30:34. doi: 10.1186/s40557-018-0240-1 (PMC5948791; doi:10.1186/s40557-018-0240-1)
Supplement: Supplementary file 1 — Physical Activity Rating (PAR) Questionnaire. (DOCX 12 kb) [file 40557_2018_240_MOESM1_ESM.docx]

Appendix 1

**Physical Activity Rating (PAR) Questionnaire**

Give yourself the appropriate PAR score (0–7) based on the following scale:

1. **Does not participate regularly in programmed recreation, sport, or physical activity.**

**0 points**: Avoids walking or exercise (for example, always uses elevators, drives whenever possible

instead of walking).

**1 point:** Walks for pleasure, routinely uses stairs, occasionally exercises sufficiently to cause heavy

breathing or perspiration.

**II. Participates regularly in recreation or work requiring modest physical activity (such as golf, horseback riding, calisthenics, gymnastics, table tennis, bowling, weight lifting, or yard work).**

**2 points:** 10-60 minute walk per week

**3 points:** Over 1 hour per week

**III. Participates regularly in heavy physical exercise (such as running or jogging, swimming, cycling, rowing, skipping rope, running in place) or engages in vigorous aerobic type activity (such as tennis, basketball, or handball).**

**4 points:** Runs less than 1.5 kilometres per week or spends less than 30 minutes per week in comparable physical activity.

**5 points:** Runs 1.5-8 kilometres per week or spends 30–60 minutes per week in comparable physical activity.

**6 points**: Runs 8-16 kilometres per week or spends 1–3 hours per week in comparable physical activity.

**7 points:** Runs more than 10 miles per week or spends more than 3 hours per week in comparable

physical activity.

Source: Jackson, A. S., et al. 1990. Prediction of functional aerobic capacity without exercise testing. *Medicine and Science in Sports and Exercise* 22:863–870. Used with permission of Lippincott,Williams &
